# Adding colchicine to immunosuppressive treatments; a potential option for biologics-refractory adult-onset Still’s disease

**DOI:** 10.1186/s13104-018-3420-8

**Published:** 2018-05-21

**Authors:** Tomoyuki Asano, Makiko Yashiro Furuya, Shuzo Sato, Hiroko Kobayashi, Hiroshi Watanabe, Eiji Suzuki, Kiyoshi Migita

**Affiliations:** Department of Rheumatology, Fukushima Medical University School of Medicine, 1 Hikarigaoka Fukushima, Fukushima, 960-1295 Japan

**Keywords:** Adult-onset Still’s disease, Colchicine, Cyclosporin A, Tocilizumab

## Abstract

**Background:**

Adult-onset Still’s disease (AOSD) is a rare inflammatory disorder characterized by the classical triad of daily spiking fever, arthritis, and typical salmon-colored rash. Resistance to first-line corticosteroids and second-line disease modified anti-rheumatic-drugs defines refractory AOSD, which mostly includes the polycyclic or chronic courses of the disease. Anti-cytokine therapies are recommended in AOSD patients who are refractory to traditional treatments. This is the first report on the efficacy of colchicine in a patient with AOSD which was refractory to immunosuppressive treatments including biologics.

**Case presentation:**

A 24-years Japanese female patient was referred to our hospital for the flare-up of AOSD under the combined treatments with steroid, immunosuppressants, and biologics. She was diagnosed with AOSD according to the Yamaguchi criteria, based on the presence of spiking fever, polyarthralgia, skin rash, and hyperferritinemia. Interleukin-6 or tumor necrosis factor-α blockade treatments were not effective, the oral administration of colchicine was stared under the immunosuppressive treatments with steroid and cyclosporine A (CyA). Colchicine treatment silenced the disease activity of AOSD. The dose of prednisolone was successfully tapered, and the elevated levels of C-reactive protein were normalized. Remission has been maintained for 13 months with the start of oral administration of colchicine.

**Conclusion:**

We concluded that colchicine is an alternative treatment in patients with refractory AOSD, particularly in those with impaired therapeutic effects against anti-cytokines therapies.

## Background

Adult onset Still’s disease (AOSD) is a rare chronic inflammatory disorder that usually accompanied with high spiking fever, arthritis and salmon pink skin rash [1]. The wide range of disease manifestations and course suggest heterogeneity of the disease entity [2]. Macrophage activation and subsequent overproduction of cytokines are involved in the pathogenesis of AOSD [3]. Therefore, cytokine-directed therapies have the potential to target macrophage-activation seen in AOSD [4]. Also, recent insights into autoinflammatory disorders have indicated that interleukin (IL)-1 blockers may be effective against steroid-refractory or immunosuppressant-refractory AOSD [5]. To treat steroid-resistant AOSD, previous reports have suggested the use of immunosuppressants such as methotrexate and cyclosporine A [6]. Additionally, anti-cytokine treatments including TNF or IL-6 blockers appear to be an efficient well-tolerated, steroid-sparing treatment against immunosuppressants-refractory AOSD [7]. The application of these biologics may provide clinicians with useful tools for the management of refractory AOSD. While targeting these cytokines has shown promising effects [7], there remain AOSD patients who do not respond to these biologics [8] and they are associated with potential severe side effects. This is the first report on the successful induction of remission with colchicine in biologics-refractory AOSD.

## Case presentation

**Fig. 1:**
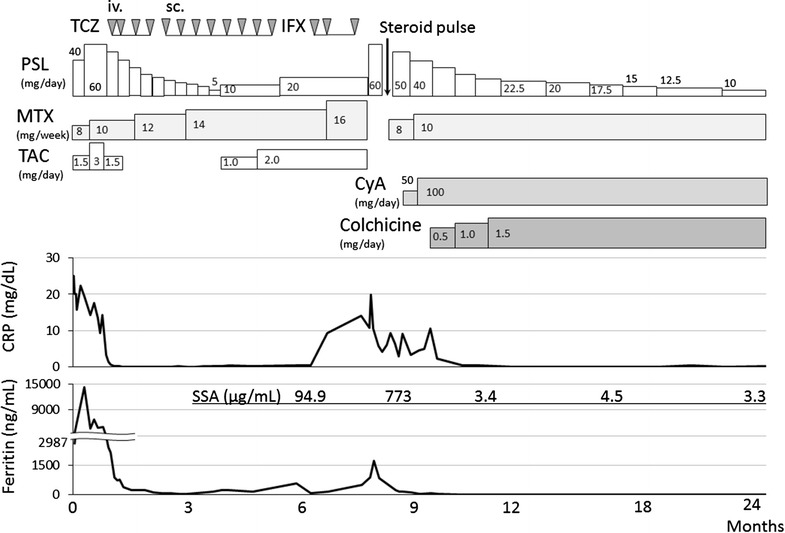
Clinical course of the patient. *PSL* prednisolone, *TAC* tacrolimus, *MTX* methotrexate, *TCZ* tocilizumab, *iv.* intravenous, *sc.* subcutaneous, *IFX* infliximab, *CyA* cyclosporine, *CRP* C-reactive protein, *SSA* serum amyloid A

24-year-old female patient was referred to our hospital because of spiking fever, arthritis in the proximal interphalangeal joints, wrists, and knees. She had been diagnosed with a systemic type of AOSD. The onset occurred 9 months previously, with acute attack of fever, bilateral tenderness and swelling over both wrists and knee joints, sore throat, and hyperferritinemia (17,900 ng/mL). The patient was diagnosed with AOSD according to the criteria of Yamaguchi et al. [9], and started on prednisolone (60 mg/day) and methotrexate (10 mg/week). However, the spiking fever was sustained, and thus tacrolimus (3 mg/day) and tocilizumab (8 mg/kg, every 4 weeks) were combined with these treatments. During that time, the patient reported some improvement. However, despite this treatment, her arthralgia increased and elevated C-reactive (CRP) was observed. Tocilizumab (8 mg/kg) was switched to infliximab (3 mg/kg), however, elevated serum levels of ferritin and CRP were sustained (Fig. 1). She had no episodes of persistent fever of unknown origin, and no symptoms such as arthritis, and skin rash that were indicative of autoimmune disease. There was no family history for autoimmune or autoinflammatory diseases.

Upon examination, inflammatory arthritis of the ankle and knee joints coinciding with the erythematous skin rash on her trunk and spiking fever (> 39 °C) were evident. Laboratory data on admission were as follows (Table 1): leukocytes, 19,000/μL (neutrophils, 90.0%); hemoglobin, 9.6 g/dL; platelets, 31.7 × 10^4^/μL; erythrocyte sedimentation rate, 41 mm (1h); C-reactive protein, 19.85 mg/dL; soluble interleukin-2 receptor, 1340 IU/L; and ferritin, 1719 ng/mL. Cytomegalovirus (CMV) antigenemia showed negative results. Urinary infection was confirmed and treated with antibiotics. Infliximab and tacrolimus were stopped, and she was treated with prednisolone and methotrexate (16 mg/week). However, her AOSD was not remitted, and hyperferritinemia was observed. Therefore, the dose of prednisolone was transiently increased to 60 mg/day and tacrolimus (TAC) was switched to cyclosporin A (CyA, 100 mg/day) because of lack of efficacy. In addition, colchicine (1.5 mg/day) was added to these treatments. The combined treatments silenced the disease activity of AOSD, and the dose of prednisolone was successfully tapered. Elevated levels of serum amyloid A (SAA; 773 µg/mL) and ferritin (1719 ng/mL) were also normalized. An analysis of the *Mediterranean fever* (*MEFV*) gene (exons 1–10) was performed by direct sequencing, but no mutation was detected. Six weeks later, the patient was discharged from the hospital. The administration of colchicine was continued and the dose of PSL was gradually tapered (Fig. 1). The patient’s clinical course remained unchanged and AOSD was successfully controlled in remission under the low-dose steroid treatment (PSL 10 mg/day) over the subsequent 15 months of follow-up (Fig. 1).

**Table 1:** Laboratory findings on admission

Peripheral blood		Serological tests	
White blood cells	19,000/μL	C-reactive protein	19.85 mg/dL
Neutriphil	90.0%	ESR (1 h)	41 mm
Lymphocyte	4.0%	sIL-2R	1340 U/mL
Monocyte	4.0%	IgG	1752 mg/dL
Eosinophil	1.0%	IgA	235 mg/dL
Basophil	1.0%	IgM	374 mg/dL
Red blood cells	4.61 × 10^6^/μL	C3	95 mg/dL
Hemoglobin	9.6 g/dL	C4	16 mg/dL
Hematocrit	32.4%	Rheumatoid factor	8.0 IU/mL
Platelet	31.7 × 10^4^/μL	ANA	< 80×
Blood chemistry		Anti-ds DNA Ab	0.6 IU/mL
Total protein	8.2 g/dL	Anti-SSA Ab	< 0.5 U/mL
Albumin	3.6 g/dL	SAA	773 µg/mL (< 8.0)
Total bilirubin	0.6 mg/dL	MMP-3	225 ng/mL (17.3–59.7)
Aspartate transaminase	48 U/L	Procalcitonin	3.89 ng/mL (< 0.05)
Alanine transaminase	11 U/L	Urinalysis	
Lactate dehydrogenase	797 U/L	Glucose	(−)
Alkaline phosphatase	224 U/L	Protein	(1+)
γ-Glutamyltranspeptidase	26 U/L	Neutrophils (sediment)	(3+)
Creatine kinase	27 U/L	Infection	
Blood urea nitrogen	12 mg/dL	HBs Ag	(−)
Creatinine	1.23 mg/dL	HCV Ab	(−)
Sodium	138 mEq/L	ASLO	275 IU/mL (< 240)
Potassium	3.8 mEq/L	Parvovirus B19 IgM	0.39 index (< 0.79)
Chlorine	99 mEq/L	CMV antigenemia	(−)
Ferritin	1719 ng/mL	Blood culture	(−)

*ESR* erythrocyte sedimentation rate, *sIL*-*2R* soluble interleukin-2 receptor, *Ig* immunoglobulin, *ANA* anti-nuclearantibody, *Anti*-*ds*-*DNA Ab* anti-double stranded deoxyribonucleic acid antibody, *SAA* serum amyloid A, *MMP*-*3* matrix metalloproteinase-3, *HBs Ag* hepatitis B virus surface antigen, *HCV Ab* anti-hepatitis C virus antibody, *ASLO* anti-streptolysin O, *CMV* cytomegalovirus

## Discussions and conclusions

Our patient is the first to be reported for effectiveness of colchicine in AOSD. The patient was treated with colchicine, and showed improvement of their clinical manifestations, such as fever and arthropathy, and normalization of their serum levels of CRP and ferritin. Although the data available to date remain limited because of the rarity of the disease, it seems clear that use of colchicine represents a good alternative to biologics therapies, which can potentially cause adverse events including infections.

The first-line therapy in AOSD is based on corticosteroid, but has various side effects [6]. Thus, immunosuppressants, such as methotrexate and cyclosporin A, have been used [10]. Recent studies demonstrated that tocilizumab treatment resulted in clinical and laboratory improvements in patients with AOSD refractory to treatment with other biologics [11]. However, in the present report, our patient was refractory to IL-6 or tumor necrosis factor (TNF)-α blockade treatments. Whereas the administration of colchicine resulted in long-term clinical remission in this case. It should be debated whether steroid pulse therapy may result in the clinical resolution of AOSD in this case. However, the effectiveness of colchicine in preventing febrile attacks was demonstrated even under the minimum dose steroid (PSL 8 mg/day) and one-course of steroid pulse therapy may not result in long-term of clinical remission of AOSD in this patient.

It could be also argued that the switch from TAC to CyA silenced the AOSD manifestations in this case. Mitamura et al., reported that CyA administration improved AOSD [12]. In contrast, Nakamura et al. reported that TAC, a calcineurin inhibitor similar to CyA, could be one of the useful option for refractory AOSD [13]. Murakami et al. reported a patient with AOSD successfully treated with TAC, while CyA was not effective [14]. Although there is no clear evidence that CyA, may be superior to TAC in the effectiveness of AOSD treatments, CyA should be comparable to TAC for treating AOSD.

Autoinflammatory diseases affect the innate immune system, and some of them are characterized by inflammasome activation and subsequent IL-1β production [15]. The clinical manifestations of autoinflammatory diseases are similar to those of AOSD, and a dramatic response to IL-1β blockade was reported in Western countries [16]. Nevertheless, IL-1β blockade treatment is not available for Japanese patients with AOSD. The finding that colchicine was effective in AOSD refractory to TNF-α or IL-6 blockade is interesting. Patients with heterozygous *MEFV* mutations with low penetrance were reported to present with clinical manifestations resembling familial Mediterranean fever (FMF) [17]. These reports indicate that the *MEFV* gene is not only associated with a single disease, FMF, but also linked to additional clinical presentations of autoinflammatory diseases [18]. The mutation analysis in our patient demonstrated no abnormalities in the *MEFV* gene. Therefore, the current case report suggests that colchicine is effective for non-FMF or non-*MEFV* gene-associated entities, because *MEFV* mutations typical to FMF were not demonstrated. Colchicine is widely used in rheumatology therapy for gout, FMF, and Bechet’s disease [19]. Its mode of action includes chemokines, and inhibition of neutrophils and endothelial cell adhesion molecules [20]. A recent investigation demonstrated that colchicine inhibits the assembly of the inflammasome complex by affecting the transport of apoptosis-associated speck-like protein containing caspase recruitment domain (ASC), an adaptor protein [21]. These findings suggest that colchicine may modulate the inflammasome-mediated proinflammatory cascades and that the broad concept of colchicine-responsive inflammatory conditions can be reconsidered. Our case report indicated that colchicine may be one of treatment options for refractory AOSD.

This is the first case report in which colchicine has been used to treat AOSD refractory to biologics treatment. Colchicine treatment resulted in clinical remission of refractory AOSD and allowed a large reduction of steroid doses in this case. Colchicine should be considered as one of the therapeutic options for AOSD refractory to anti-cytokines treatment.

## Abbreviations

AOSD: adult onset Still’s disease
CyA: cyclosporin A
DMARDs: disease modified anti-rheumatic-drugs
FMF: familial Mediterranean fever
IL-6: interleukin-6
TAC: tacrolimus
TNF-α: tumor necrosis factor-α
